# Role of human innate lymphoid cells in IMID

**DOI:** 10.1186/1479-5876-10-S3-I16

**Published:** 2012-11-28

**Authors:** Hergen Spits, Jochem Bernink, Charlotte Peters, Jenny Mjosberg

**Affiliations:** 1 Tytgat Institute for Intestinal and Liver Research, Academic Medical Center at the University of Amsterdam, The Netherlands

## 

Innate lymphoid cells (ILC) are immune cells that lack a specific antigen receptor, yet possess the capacity to produce an array of effector cytokines that in variety matches that of T helper cell subsets. Innate lymphoid cells function in lymphoid organogenesis, tissue remodeling anti microbial immunity and inflammation particularly at barrier surfaces. The ability of ILCs to promptly respond to insults inflicted by stress causing microbes strongly suggests that ILC are critical in first line immunological defenses. In addition ILC are involved in repair of tissue damage inflicted by pathogenic microbes. In my presentation I will present data on developmental requirements, lineage relationship and effector functions of members of two families of innate lymphoid cells: Rorgt-expressing cells involved in lymphoid tissue formation, mucosal immunity and inflammation and Type 2 innate lymphoid cells that are important for helminth immunity. In addition I present evidence for the existence of a novel ILC population that is dedicated to produce interferon gamma. I will also discuss the possible roles of ILC in pathology of immunity mediated inflammatory and infectious diseases including allergic diseases.

